# Effect of Residual Cuts on Deactivation of Hierarchical Y Zeolite-Based Catalysts during Co-Processing of Vacuum Gas Oil (VGO) with Atmospheric Residue (ATR)

**DOI:** 10.3390/molecules29194753

**Published:** 2024-10-08

**Authors:** Jayson Fals, Esneyder Puello-Polo, Edgar Márquez

**Affiliations:** 1 Grupo de Investigación en Oxi/Hidrotratamiento Catalítico y Nuevos Materiales, Programa de Química-Ciencias Básicas, Universidad del Atlántico, Barranquilla 080003, Colombia; 2 Departamento de Química y Biología, Grupo de Investigación en Química y Biología, Facultad de Ciencias Básicas, Universidad del Norte, Barranquilla 080003, Colombia

**Keywords:** atmospheric petroleum residue, FCC catalyst, spent catalysts, catalytic cracking, deactivation, coke

## Abstract

The influence of residual cuts on the deactivation of hierarchical Y zeolite-based catalysts during the co-processing of vacuum gas oil (VGO) with atmospheric residue (ATR) was investigated. The experiments were conducted in a laboratory-scale MAT-type reactor. The conversion of VGO, ATR, and their 70:30 (mass basis) mixture was examined using two composite catalysts: Cat.Y.0.00 and Cat.Y.0.20. The operating conditions closely resembled those of the commercial catalytic cracking process (550 °C and contact times of 10 to 50 s). When ATR was processed individually, the conversion remained below 50 wt%. However, significant improvements in conversion rates were achieved and catalyst deactivation was mitigated when ATR was co-processed with VGO. Notably, the BET surface area and average mesopore volume were adversely impacted by ATR, which also led to the accumulation of high levels of metals and nitrogen on the spent catalyst, detrimentally affecting its acidic and structural properties. Moreover, substantial coke deposition occurred during ATR cracking. The soluble and insoluble coke analysis revealed H/C ratio values of up to 0.36, indicative of polycondensed coke structures with more than ten aromatic rings. The nature of the coke was confirmed through TPO and FTIR analyses. Interestingly, the CatY.0.20 catalyst exhibited less activity loss, retaining superior acid and structural properties. Co-processing Colombian atmospheric residue with ATR loadings of 30 wt% (higher than the typical 20 wt%) in catalysts formulated with hierarchical zeolites presents a promising alternative for commercial applications. This research opens avenues for optimizing catalytic cracking processes.

## 1. Introduction

Currently, the oil industry faces the challenge of converting heavy hydrocarbons into lighter products of higher commercial value through fluidized catalytic cracking (FCC) [1,2,3]. In this context, zeolites continue to be the predominant materials in the formulation of catalysts due to their notable catalytic activity, which is due to the acidic characteristics of the Brønsted and Lewis sites present in their structure [4,5]. However, the effectiveness of zeolites is limited by their pore dimensions, which are inadequate for processing large molecules present in atmospheric residue and other heavy feedstocks [6,7,8]. With the increase in oil exploration, the density of crude oils has become increasingly heavier, presenting an additional challenge for traditional catalysts. These heavy crude oils contain compounds that exceed the pore size of the zeolites, which limits diffusion and accessibility to the active sites of the catalyst [9,10]. Thus, the need to develop and employ new catalytic materials with larger and improved pore structures is crucial to optimize the processing of these heavy feedstocks and meet the growing demand in the industry. 

The oil industry is currently facing significant challenges due to fuel oil depletion, the high cost of crude oil, and the need to maximize the efficiency of refining operations. This situation has led to the use of residual hydrocarbon cuts in commercial catalytic cracking units. A key strategy to address these challenges is the incorporation of residue into conventional feedstocks, such as vacuum gas oil (VGO) [11]. These residues include a wide variety of feedstocks, such as atmospheric distillation tower bottoms (ATR), vacuum distillation bottoms (VTR), deasphalted oil, and aromatic extracts [12]. Although the inclusion of residue in FCC feedstocks is not a new strategy, its application has evolved over time. Currently, the proportions of residue mixed with VGO have increased significantly. Among the various options available for converting residue into higher-value products, such as hydrocracking, coking, solvent deasphalting, and FCC, the latter emerges as one of the most effective and economical solutions in many cases. The adaptability of the FCC to process these residues and convert them into useful products underlines its relevance and advantages in optimizing hydrocarbon production in the current context [13,14].

The residue is distinguished from conventional feedstocks by its higher content of catalyst-contaminating metals, such as Ni, V, Na, and Fe, as well as its high content of polyaromatic species that are strong coke formers. In addition, these residues contain sulfur and nitrogen heteroatoms, which contribute to SO_x_ and NO_x_ emissions and the formation of sulfurous hydrocarbons in the products. The residual components have a high concentration of naphthenics, aromatics, resins, and asphaltenes, and their chemical structure is extremely complex [15,16,17]. In this sense, catalysts must obey a series of conditions, such as greater tolerance to contaminating metals, resistance to coke, and minimum mass transfer limitations between particles. Under these conditions, and also considering the operating conditions, a given catalyst will not be optimal in many cases; therefore, custom-made catalysts are needed [18,19,20].

An effective alternative to counteract diffusion problems in catalysts is the modification of the porous structure of zeolites through alkaline treatment. This methodology has been widely documented in the literature, showing significant improvements in the catalytic performance of Y zeolites in cracking reactions [21]. Previous studies have shown that the generation of mesoporosity in zeolites not only improves resistance to coke deactivation but also increases tolerance to contaminating metals and reduces the mass transfer limitations of reactants and products. Silvia et al. studied the cracking of 4-propanol on hierarchical zeolite MCM-22 catalysts and found that the greater mesoporosity generated in the zeolite favoured resistance to deactivation by coke [22].

Atmospheric residue is an extremely heavy and complex feedstock that contains high levels of contaminants, such as metals and nitrogenous compounds, which cause the rapid deactivation of catalysts [23]. These properties negatively affect process efficiency, increasing operating costs and reducing profitability. To address this problem, it is necessary to optimize the conditions of ATR co-processing with lighter feedstocks, such as vacuum gas oil (VGO), and develop custom-formulated catalysts. To enhance the durability and minimize the deactivation of catalysts under the harsh conditions imposed by heavy residues, this study aims to investigate the impact of residual feedstocks, particularly atmospheric residue (ATR), on the deactivation of catalysts formulated from hierarchical zeolites. This investigation will consider both individual and co-processing with vacuum gas oils (VGOs). This study will examine how contaminating metals, nitrogen, and coke affect the acidic and structural properties of the catalysts. By analyzing spent catalysts, we aim to identify deactivation mechanisms and propose strategies to optimize the durability and efficiency of the FCC process. The findings are expected to contribute to the development of methods that improve the economic balance of the process and increase flexibility in the use of feedstocks in the oil industry, ultimately providing a foundation for formulating more resilient and efficient catalysts for heavy residue conversion.

## 2. Results and Discussion

### 2.1. Feedstocks

Characterizing the feedstock utilized in the catalytic cracking process yields crucial insights into the hydrocarbons comprising them, as well as the presence of contaminating metals or impurities that may impact catalyst activity. Table 1 summarizes the properties of the feedstocks used in this study, as determined through various analytical techniques. Among the various properties, API density serves as a prominent indicator of quality. Based on their API gravity values, these feedstocks can be categorized as light (API > 31.1), medium (22.3 < API ≤ 31.1), and heavy (API ≤ 22.3) [24], as observed in both VGO and ATR residue. The lower API values exhibited by the residue were expected, taking into account the high content of asphaltenes and resins in this cut (>50 wt%). 

Conradson carbon content is a reliable index of aromatic species content; a high value (>5 wt%) indicates that the residual cut is very rich in aromatic species, as was the case for ATR, whose value was 5.9 wt%. On the other hand, if values less than 1 are present, this indicates that the cut may be made up mostly of n-paraffins [25,26]. The lower CCR value observed in VGO was consistent with its higher proportion of n-paraffins (FS = 47.4 wt%, see Table 1). Furthermore, the CCR content was directly related to the API density value, since high API density is an indicator of low potential for generating Conradson carbon residue.

It is widely known that FCC catalysts are poisoned and deactivated by the presence of metals in feedstock [27,28]. In crude oil and its residual cuts, it is common to find various groups of metals suspended in the form of salts or in the form of organometallic compounds. Within this group of metals, vanadium, nickel, and iron are the main ones responsible for the loss of activity. Of the two feedstocks, the ATR had a higher content of these metal species, which made it a harmful feedstock for the catalyst. For example, vanadium had the highest concentration of 4.22 ppm, with this metal being the most harmful in the process. For its part, the nitrogen that was present in a greater proportion in the ATR (N = 1.40 wt%) could poison the active sites of the catalyst, especially the acid sites. Therefore, a greater impact of the residual feedstocks on the deactivation of the catalyst was expected [29].

The simulated distillations of each feedstock are also presented in Table 1. It was observed that the boiling temperatures for the products distilled from the atmospheric residue required higher temperatures to obtain the different distillation fractions. In total, 10% of its liquid products were distilled at 411 °C, 50% at 490 °C, and 70% at 518 °C, while the temperatures corresponding to these percentages for VGO were 387 °C, 450 °C, and 487 °C, respectively. This indicated a greater presence of light hydrocarbons in the VGO. The boiling points obtained from the simulated distillation curves had a direct correlation with their API density values; a higher API density value indicated a greater amount of light hydrocarbons, as was the case for VGO.

### 2.2. Catalyst Properties

The most important properties of the catalysts used in this work are shown in Table 2. Table 2 shows the textural, crystalline, and acidic properties of the composite catalysts. The properties of both catalysts differed significantly, given that they were prepared using hierarchical zeolites with different degrees of mesoporosity. The most significant differences in the textural properties of catalyst CatY.0.20 (lower specific surface area and micropore contribution and higher mesopore diameter and mesopore volume) compared to those of catalyst CatY.0.00 supported in part their expected performances. 

Indeed, the CatY.0.20 catalyst favored the processing of feedstocks with a high proportion of residue, which included molecules with a larger molecular size, facing more severe diffusion restrictions in the catalyst pore system. In this sense, a greater contribution to the pore volume of larger mesopores was especially appropriate, as confirmed by the BJH method. The zeolite content was according to the catalyst formulation, around 35 wt%. These values differ slightly from the typical values of FCC equilibrium catalysts, which are normally close to 20 wt% due to the wear they suffer during the multiple deactivation/regeneration cycles [30].

The Brønsted acid sites in catalysts have greater participation in cracking reactions than the Lewis sites [31,32]. Therefore, a catalyst with high Brønsted acidity will probably have high activity and favor cracking reactions. The CatY.0.20 catalyst showed a higher concentration of Brønsted acid sites. The cracking reactions occurred at the Brønsted sites of the catalyst through two pathways: protolytic cracking via the five-coordinated carbonium ion and cracking via the three-coordinated carbenium ion [33]. The different reactions that develop during catalytic cracking processes require sites with different acid strengths. For example, cracking reactions require sites with strong acidity, while others, such as hydrogen transfer reactions, require sites with weaker acidity [33]. Given the bimolecular nature of hydrogen transfer reactions, they are favored by a higher density of acid sites, as this increases the probability of paired acid sites existing. On the contrary, a greater proportion of isolated acid sites resulting from a lower density of sites will preferentially favor cracking reactions, which are unimolecular in nature [34,35]. Of the two catalysts used, CatY.0.20 showed a greater number of strong acid sites, which is why it had better performance in the cracking reactions. However, it also presented the highest density of total acid sites that, in turn, could favor hydrogen transfer reactions that give rise to carbonaceous deposits.

### 2.3. Catalytic Performance

The catalytic performance of the hierarchical Y zeolite-based catalysts was evaluated under experimental conditions adjusted according to the objectives set out in this work in a MAT reactor (see Section 3.3). In previous research, the co-processing of atmospheric residue (ATR) with VGO has been reported using equilibrium catalysts supplied by refineries and Y zeolites [14,36]. However, these studies were limited to analyzing the impact of incorporating residual cuts on yields and product distribution. The importance and novelty of our research lies in the fact that, for the first time, the effect of catalyst deactivation due to the deposition of coke (nature of coke), metals (poisons), and nitrogen, as well as its impact on the acidic and structural properties of the catalyst, is here investigated. Additionally, our research group synthesized these catalysts using hierarchical zeolites obtained by alkaline treatment, which provides significant innovation in the field.

#### Conversion and Product Distribution

**Figure 1:**
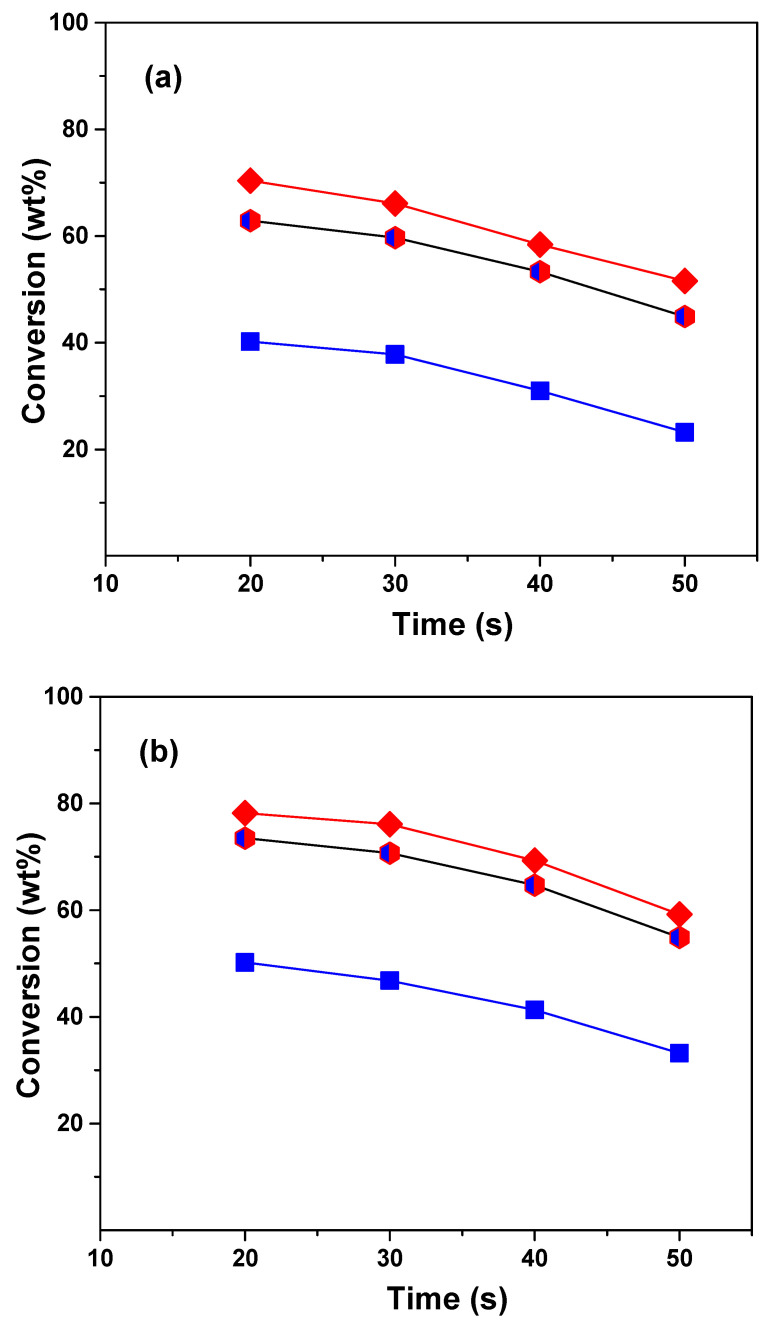
Conversion of VGO (*red diamonds*), ATR (*blue squares*), and VGO:ATR (*blue and red hexagon*) with catalysts CatY.0.00 (**a**) and CatY.0.20 (**b**).

The conversion was calculated as the sum of the yields of dry gas, liquefied petroleum gas, gasoline, and coke cuts. Figure 1 shows the conversions obtained in the cracking of VGO, ATR, and the VGO:ATR mixture over the CatY.0.00 and CatY.0.20 catalysts as a function of the reaction time at 550 °C. The obtained profiles showed typical behaviors, with conversion decreasing as a function of reaction time, as was expected for a microactivity test (MAT) unit with a fixed bed [37]. In our study, we observed significant differences in the conversion of the feedstock and their mixture. The atmospheric residue showed the lowest conversion values, reaching only 20 wt% with the CatY.0.00 catalyst. However, co-processing with VGO significantly improved the conversion, reaching a performance close to that of the VGO, with the highest concentration in the mixture (70 wt%). The co-processing of ATR with VGO significantly improved conversion compared to the processing of ATR alone as a result of the synergistic effect of the co-feed. VGO is a mixture of lighter hydrocarbons with higher H/C ratios compared to ATR, which contains more heavy fractions and aromatics. By combining the two, VGO can help hydrogenate or stabilize certain reactive intermediates in ATR, reducing coke formation and allowing for the more efficient cracking of the heavy compounds in ATR. Among the two catalysts evaluated, the one prepared with the hierarchical zeolite with the highest mesoporosity demonstrated the best performance with the three feedstocks. The CatY.0.20 catalyst was distinguished not only by its higher mesoporosity but also by a higher concentration of strong Brønsted acid sites. These strong acid sites are the main promoters of cracking reactions, which result in a higher product yield and, consequently, higher conversion values. This catalyst achieved conversions close to 50 wt% with the atmospheric residue, evidencing its better performance in terms of activity and efficiency. In terms of conversion, the CatY.0.20 catalyst is a good choice for processing residue from the atmospheric refining tower.

**Figure 2:**
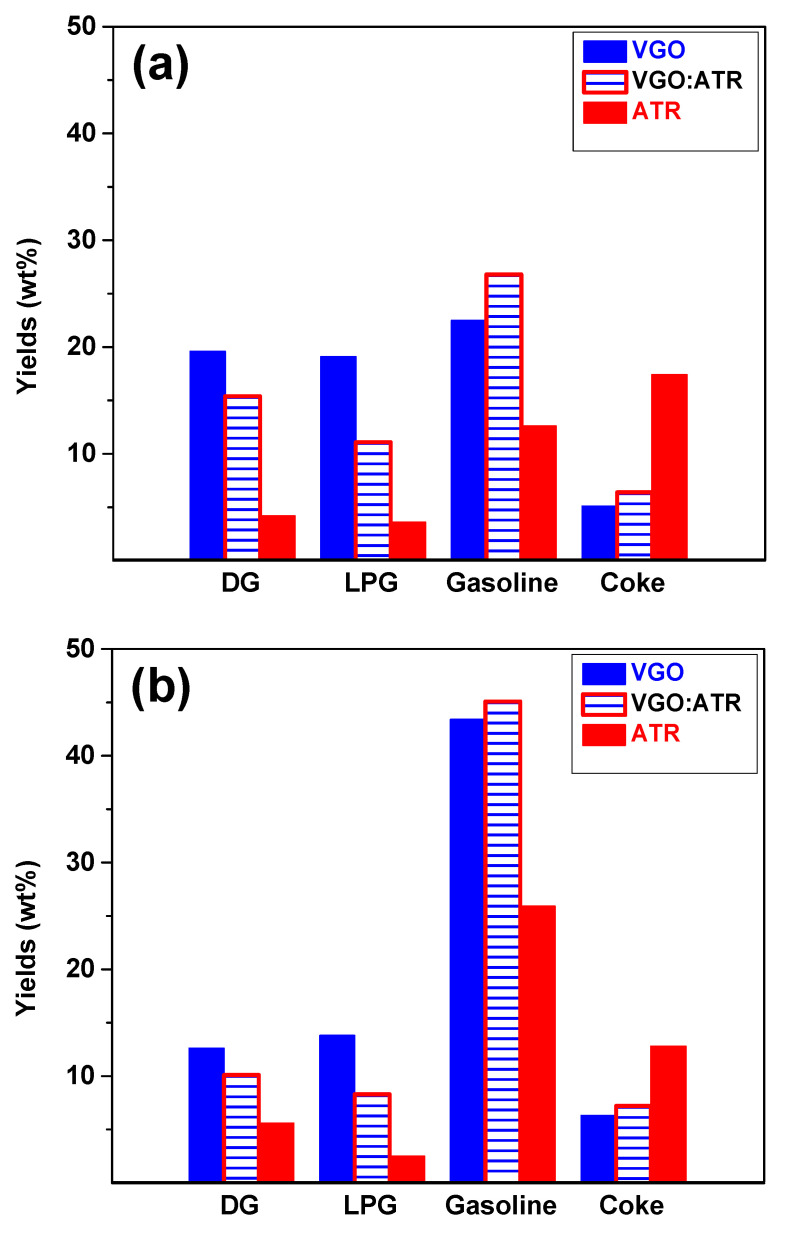
Yields of products obtained from cracking VGO, ATR, and VGO:ATR with CatY.0.00 (**a**) and CatY.0.20 (**b**) over 30 s.

The product distributions from the conversion of the feedstocks at a reaction time of 30 s are shown in Figure 2. When evaluating the performance of both catalysts with the three feedstocks, it was observed that gasoline was the main product, reaching yields greater than 40 wt% with CatY.020. This high performance is very positive since current refining processes are oriented towards the production of liquid fuels, given the high demand for gasoline in the global energy market [38]. The CatY.0.20 catalyst, with its higher mesoporosity and higher concentration of Brønsted strong acid sites, showed superior performance in converting feedstocks, favoring the formation of products in the gasoline range. The presence of these strong acid sites is crucial, as they promote the cracking reactions necessary to break down larger molecules into smaller, more valuable fractions [39]. These results highlight its potential as a highly efficient and versatile material in refining processes with atmospheric residue, which is significantly superior compared to other catalysts evaluated [14,15]. The residues, due to their high composition of resins and asphaltenes, showed low gasoline production when processed independently. However, when co-processed with VGO, a significant improvement in gasoline production was observed due to synergistic effects, even exceeding the production obtained with pure VGO. The synergistic effect improved conversion and product yields in the gasoline range. This occurred because VGO, with its higher H/C ratio, facilitated the stabilization of radicals and intermediate products generated during the cracking of the heavy fractions of the ATR, allowing for greater decomposition of these molecules into light hydrocarbons. In this way, the cracking of the heavier components of ATR was optimized, resulting in an increase in both gasoline production and quality. This phenomenon highlights the importance of co-processing to improve refining yields.

The results also showed that the performance regarding gases (DG and LPG) was favored by the higher microporosity of the catalyst. In this sense, the CatY.0.00 catalyst, characterized by its high microporosity, showed the best performances in gas production. Of the different feedstocks studied, VGO was the most selective towards gas production, reaching values of up to 20 wt%. The high microporosity of the CatY.0.00 catalyst eased the formation and release of gases due to its ability to promote the cracking reactions of the terminal chains present in the bulky molecules. This behavior was consistent with the nature of VGO, which has more paraffin components and greater crackability in the presence of a highly microporous catalyst.

As for coke, as expected, the atmospheric residue (ATR) showed the highest selectivity towards this product due to the high concentration of asphaltenes and resins in its composition. This trend was observed more strongly in the CatY.0.00 catalyst, indicating a greater impact on catalyst deactivation. In principle, coke is considered an undesirable product, as it contributes to the loss of catalytic activity and reductions in catalyst lifetime [40]. However, it plays a significant role in energy balance in the process. Catalytic cracking, being a cyclic deactivation and regeneration process, requires elevated temperatures to burn off the accumulated carbon deposits [41]. The coke produced during the process provides the fuel needed to reach these regeneration temperatures, thus improving the thermal efficiency of the process. In this sense, although coke formation must be controlled to avoid premature catalyst deactivation, its presence is essential for the energy balance of the process. A detailed study of coke, including its nature and composition, will be addressed in greater depth in Section 2.3.

**Figure 3:**
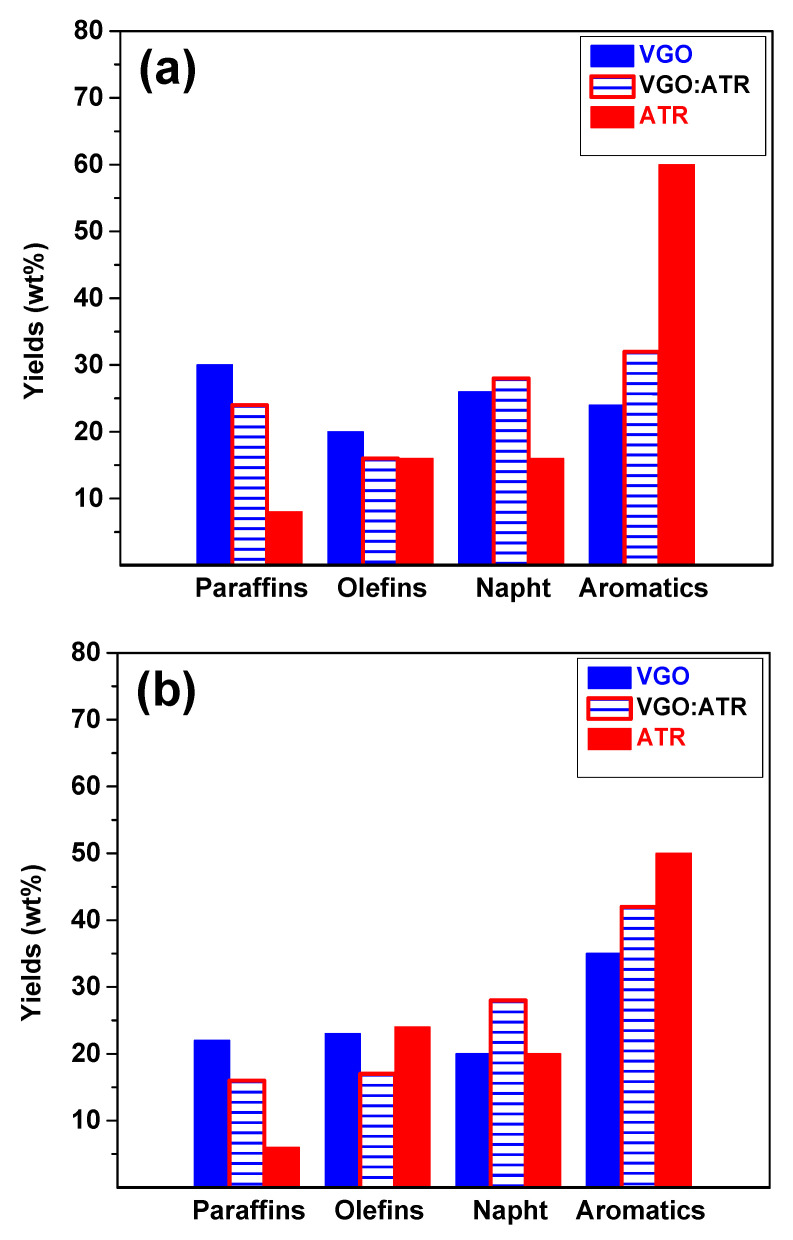
Composition and quality of gasoline cut obtained from cracking VGO, ATR, and VGO:ATR with CatY.0.00 (**a**) and CatY.0.20 (**b**) over 30 s.

Refining processes are not only aimed at producing liquid fuels but also at ensuring fuel quality. Figure 3 shows the results of the gasoline cut composition at a reaction time of 30 s, where a greater presence of aromatic and olefin species was observed when using the CatY.0.20 catalyst. This composition was directly associated with the octane number (Research Octane Number, RON), which implied a significant improvement in the quality of gasoline obtained with this catalyst. The greater mesoporosity and acidity of CatY.0.20 facilitated the formation of aromatic and olefinic compounds, which are crucial for increasing the RON and, therefore, fuel efficiency in internal combustion engines [42]. The RON values obtained for the different feedstocks in the CatY.0.20 catalysts were as follows: VGO:ATR blend (84), ATR (83), and VGO (81). For the CatY.0.00 catalyst, the RON values were VGO:ATR blend (82), ATR (83), and VGO (79). These results highlight the improvement in cut quality when co-processing residue.

### 2.4. Characterization of Coked Catalysts

**Figure 4:**
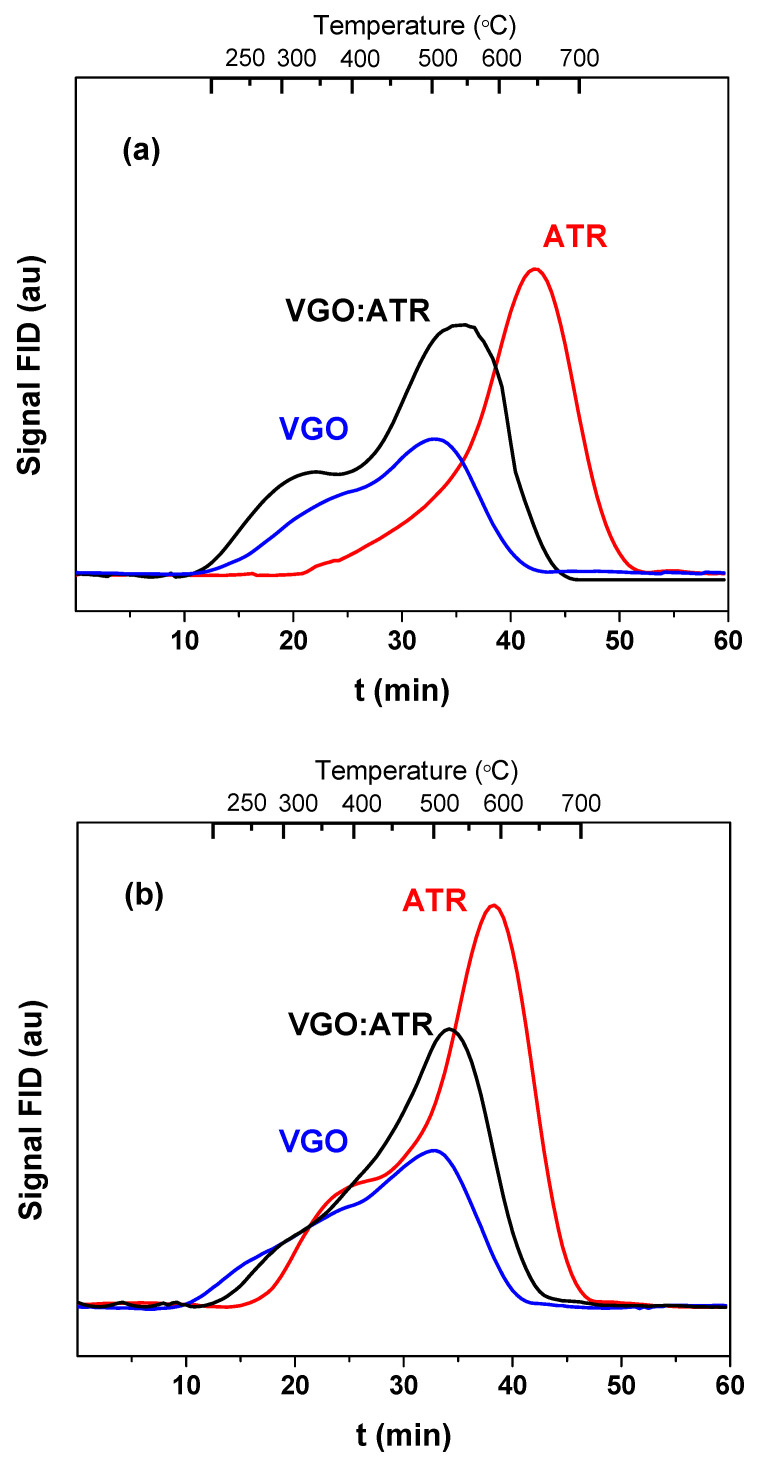
Combustion profiles (TPO) of the coke formed in the cracking of VGO with CatY.0.00 (**a**) and CatY.0.20 (**b**) over 30 s.

The properties of the coke deposited on the surface of the catalyst during the coprocessing of vacuum gas oil with atmospheric residue were studied using different analytical techniques. The amount and nature of the coke deposited on the catalysts were determined using the programmed temperature oxidation technique. Figure 4 shows the TPO profiles of the coke deposited on the spent catalysts with a reaction time of 30 s. As observed in Figure 4, the amount of coke produced with the ATR cut was higher than the other feedstock, which generated a negative impact on the catalyst activity. It is known that carbonaceous deposits play a key role in the energy balance of catalytic cracking processes, given that they are cyclical (deactivation/regeneration), which makes their production necessary. However, very high values affect the economics of the process [42]. The TPO profiles not only allowed us to quantify the coke but also provided information on the type of coke deposited. A shift at higher combustion temperatures is an indicator that the deposited coke is more difficult to oxidize, may be trapped in the micropores of the catalyst, and may also have a high basic character that interacts with the strong sites of the catalyst, causing shifts at higher temperatures. In this sense, the atmospheric residue not only favored the greater production of coke but, also, its combustion temperatures were the highest, higher than 580 °C, which, in principle, could indicate the formation of a more concentrated and dense coke of greater basic character. This effect was more representative in the catalyst with greater microporosity (CatY.0.00), whose maximum combustion peak was observed at a temperature of 650 °C. When co-processing VGO with ATR, there were synergistic effects on the formation and characteristics of the coke. The addition of VGO reduced its formation and the coke-burning temperatures, whose observed values were lower than 550 °C. The greater proportion of VGO (rich in paraffin) in the mixture acted as a diluent medium, reducing the concentration of heavy coke precursors in the reactive phase and decreasing the rate of the polymerization and condensation of these precursors in the catalyst. This behavior favored the formation of less condensed and more dispersed coke structures that burned more easily. Of the three feeds used, as expected, VGO presented combustion peaks at lower temperatures (around 500 °C). In general, with all the feedstock and with both catalysts, two peaks appeared: one at low temperatures, typical of coke with a lower degree of condensation, and the one already known at higher temperatures and characteristic of residual feedstock.

Coke was also studied by FTIR spectroscopy; the aromatic compounds showed stretching and deformation bands at 1580 cm^−1^, while the olefinic compounds presented stretching and deformation bands associated with the C=C double bonds at 1610 cm^−1^, signals that have been widely studied by our research group and other authors. Table 3 shows the relative intensities of the bands at 1580 and 1610 cm^−1^ observed from the FTIR spectra of coked catalysts at a reaction time of 30 s. The two types of coke were identified in all coked catalysts; however, some notable differences were observed. ATR led to the highest intensities of aromatic coke for both catalysts, its intensities tripling those of olefinic coke. These two techniques complemented and confirmed each other; the high combustion temperature of ATR observed in the TPO profiles was related to its high aromaticity and condensation, which resulted in a coke structure that was more difficult to oxidize. For its part, the lower combustion temperature of VGO was related to its lower aromaticity and greater presence of aliphatic structures, which resulted in a coke structure that was easier to oxidize.

The soluble and insoluble coke was separated, dried (after eliminating the HF-containing solution), and characterized by elemental analysis. Table 4 shows the atomic H/C ratio of different hydrocarbons. For both catalysts, only 15 wt% of the coke could be extracted with chloroform. The soluble coke was generally the lightest fraction of the coke, having most compounds trapped in the micropores of the zeolite, while the insoluble coke, or heavier fraction of the coke, corresponded to that deposited in the meso- and macropores of the matrix. Despite everything, this result is indicative and depends on the compositions of coke fractions. The structures found by gas chromatography were in the H/C ratio range between 0.7 and 1.4, which corresponds to aromatic species with between one and four rings. Species with a higher degree of condensation (H/C < 0.7) are insoluble in dichloromethane and require another analysis technique. The VGO and VGO:ATR mixture favored a soluble coke with an H/C ratio between 0.9 and 1.4, which corresponded to one- and four-ring aromatic species that have been previously identified and reported by our research group [21]. In the case of the residue consisting mainly of aromatics, an insoluble coke with a higher degree of condensation was favored, composed mainly of species with an H/C ratio between 0.7 and 0.9. Of the two catalysts used, CatY.0.00 exhibited species with a higher degree of condensation, which suggests that the coke may have been trapped in the pores of the zeolite, thus favoring its polymerization.

Table 5 shows the elemental analysis of the insoluble coke deposited on both catalysts with the different feedstocks at a reaction time of 30 s. The elemental analysis carried out on the CatY.0.00 and CatY.0.20 catalysts suggested that the insoluble coke deposited contained condensed aromatic structures of more than five rings and low H/C ratios, with values of up to 0.36. Other authors have investigated the nature of the coke deposited on the surface of zeolites and zeolitic catalysts. Table 5 shows some coke structures identified in the spent materials and their H/C ratios. Based on these investigations, it was confirmed that the insoluble coke present in the spent catalysts studied in this research had voluminous structures, with the presence of polyaromatic rings with more than ten members. Of the three feedstocks, ATR favored the formation of coke with a higher degree of condensation. The H/C ratio of the insoluble coke with the VGO was between 0.57 and 0.59, and, hence, the coke must have had a lower degree of condensation compared to the coke deposited in the ATR and the mixture. For its part, the coprocessing of VGO with ATR showed an H/C ratio close to that observed with VGO, with a higher composition in the mixture. It is important to highlight that coke with a lower H/C ratio generates a negative impact on catalyst activity due to the greater difficulty of combustion during the deactivation/regeneration process.

Nitrogen present in hydrocarbons entering catalytic cracking is considered a poison for catalysts since they can adsorb on the active sites of the catalyst, especially Brønsted acid sites. This adsorption blocks the active sites, reducing the efficiency of the catalyst in the process. The presence of nitrogen affected the selectivity of the products obtained, decreasing the production of the most valuable products such as gasoline and increasing the formation of unwanted products such as coke. A comparison of the feedstocks shows that the residue, having the highest concentration of nitrogen, favored the highest production of coke and rapid deactivation of the catalyst. This shows that molecules with nitrogen are important coke precursors and are preferentially retained. Shi et al. studied coke deposited on FCC catalysts and identified three types of N species: pyrrolic N, pyridinic N, and quaternary N [23]. The first two were unstable and were found in the outer layer of the coke, while the quaternary N was stable. Of the two catalysts used in the conversion of hydrocarbons, Cat.Y.0.20 favored a higher concentration of coke, a behavior that was due to the greater presence of strong acid sites compared to Cat.Y.0.00. To eliminate the retained nitrogen species, the catalyst needed to be subjected to high temperatures.

### 2.5. Characterization of Spent Catalysts

Table 6 shows the metal concentrations and textural and acidic properties of the coked catalysts at a reaction time of 30 s. In all cases, both catalysts suffered a reduction in BET area and average mesopore diameter due to coke accumulation, thus reducing accessibility to the catalyst surface. On the other hand, coke deposits could also narrow existing pores, reducing the average mesopore diameter. ATR was the feedstock that most affected the catalyst properties, causing losses of more than 60% of the BET area in CatY.0.00 and up to 55% in CatY.0.20. The average mesopore diameter also suffered a reduction, with losses close to 50% in both catalysts. The presence of metals such as nickel, vanadium, and iron could deposit on the active sites of the catalyst, poisoning these sites and clogging the pores. These metals could form oxides that occupied space within the catalyst pores, contributing to the loss of surface area and pore volume. For example, vanadium had the highest concentration of 4.22 ppm, being the most harmful metal in the process, since it not only enhanced the hydrogen transfer reactions but also, during the catalyst regeneration process, underwent a hydrolysis reaction that gave rise to vanadic acid, which destroyed the zeolitic structure, causing irreversible damage to the catalyst. This phenomenon was primarily manifested in the physicochemical characteristics of the catalyst, encompassing a reduction in surface area, dealumination, crystallinity, and the depletion of Brønsted acid sites [43,44,45]. 

Concerning the acidic properties, both Brønsted and Lewis acid sites were significantly impacted by coke deposition. The residue caused acidity losses of up to 70% in Brønsted sites and up to 50% in Lewis sites. These findings indicate a higher selectivity of coke, metals, and nitrogen for the more exposed Brønsted sites on the catalyst surface. While nitrogen compounds do not physically obstruct pores like coke and metals, they can alter the chemical properties of the internal surface, thereby affecting the material’s texture. This degradation adversely affects catalyst efficiency, necessitating the regeneration or replacement of the catalyst to maintain process performance, as is commonly practiced in commercial FCC units [46]. 

## 3. Materials and Methods

The experiments of catalytic conversion were carried out using three different feedstocks: a vacuum gas oil, an atmospheric residue, and a 70:30 mass mixture of these (VGO:ATR). The catalysts used were two catalysts with typical FCC catalyst formulations (CatY.0.00 and CatY.0.20), whose properties are shown in Table 2. The powdered catalysts used in this research were prepared with a composition typical of catalysts used in fluidized bed catalytic cracking (FCC) processes. These catalysts consisted of 35 wt% of zeolite Y, 45 wt% of matrix, and 20 wt% of binder. The shape of the catalysts was spherical and the exact methodology used to prepare these catalysts has been detailed in previous work by our research group, which is available for consultation [21]. 

### 3.1. Feedstock Characterization

The vacuum gas oil and atmospheric residue used in this investigation were supplied by a Colombian refinery. The main properties of the VGO and ATR are shown in Table 1. The feedstock properties were determined by means of the following techniques. API gravity was measured following ASTM D287-12b [47]. The Conradson carbon residue (CCR) present in the hydrocarbon samples was determined following the ASTM D4530-15 standard [48]. The simulated distillation curves were obtained in a Shimadzu GC-2014 gas chromatograph equipped with an FID, with a non-polar column of 30 m with a diameter of 250 μm and a film thickness of 0.25 μm, following the ASTM D2887-23 standard [49]. The different groups of hydrocarbons present in the VGO and ATR were separated into four fractions (saturated, aromatic, resin, and asphaltene, SARA), following the procedure described in the ASTM D2007-11 standard [50]. The amounts of Ni, V, Na, and Fe were determined using Perkin-Elmer Optima 7300 DV spectrometer inductively coupled plasma optical emission (ICP-OES). Before preparing the VGO:ATR mixture, the given ATR was kept in a hot bath at a slightly elevated temperature (around 40–50 °C) to ensure that it remained in a liquid state, allowing it to be manipulated and mixed with the VGO. The mixture (VGO:ATR) was obtained by mixing the masses of each component at a 70:30 ratio. 

### 3.2. Catalyst Characterization 

The catalysts were characterized using various analytical techniques. The textural properties were determined by nitrogen adsorption using a 3-Flex™ automatic analyzer from Micromeritics. The samples (fresh and coked) were previously degassed at 300 °C for 24 h under vacuum conditions of 10^−6^ mmHg, while the adsorption–desorption data were obtained at T = −196 °C. The specific surface areas were calculated using the Brunauer–Emmett–Teller (BET) model, with the pore volume determined from the quantity of adsorbed nitrogen at a relative pressure of P/P0 ~ 0.98. The average mesopore diameters and pore size distributions were determined by applying the Barrett–Joyner–Halenda (BJH) model. The mesopore-specific surface area and micropore volume were estimated using the t-plot method.

The crystallographic properties were determined by means of X-ray diffraction with a Siemens D500 diffractometer with a CuKα monochromatic radiation source (λ = 1.5418 Å). Data were obtained in the 2θ range of 5–50° at a step size of 0.05° and a dwell time of 3 s per step. The crystallinities and unit cell sizes were calculated according to the ASTM D-3906-03 [51] and ASTM D-3942-19 [52] standards, respectively.

The acidic properties (nature, strength, and density of acid sites) of both catalysts (fresh and coked) were studied by means of Fourier transform infrared spectroscopy FTIR, using pyridine as a probe molecule. Wafers with a diameter of 15 mm were prepared with 80 mg of zeolite using a pressure of 4 tons/cm^2^. The samples were degassed at a temperature of 450 °C for 2 h and 10^−3^ Torr; then, the background spectrum was recorded after cooling to room temperature. The pyridine molecules were adsorbed at room temperature and, after successive desorptions (150, 300, and 400 °C), spectra were gathered at 25 °C and 10^−3^ Torr. The spectra were obtained at a resolution of 4 cm^−1^ and 256 scans with a Nicolet FTIR spectrophotometer. The absorption FTIR bands at 1540 and 1450 cm^−1^ were identified as the acid Brønsted and Lewis sites by the pyridinium ion and pyridine formation, respectively. The amount of pyridine that remained adsorbed after desorption at 150 °C accounted for the total (weak + medium + strong) of acid sites, the amount retained after desorption at 300 C represented the sum of medium and strong acid sites, and, after desorption at 400 °C, strong acid sites were determined [53]. 

### 3.3. Catalyst Evaluation Tests 

The experiments of catalytic cracking were carried out using a micro activity test (MAT) unit with a fixed-bed reactor containing 3.0 g of catalyst, with the mass flow rate of VGO being 2.0 g/min, operated to mimic a series of FCC cycles. Before the experiments, the catalysts were heated under an air stream of 570 °C for 30 min. Both the feedstock and the amount of catalyst were kept constant, establishing the contact time as an operating variable, to vary the C/O ratio. The reactions were performed at 550 °C and the reaction times were 20, 30, 40, and 50 s. The products generated in the process were collected at the outlet of the reactor and subsequently analyzed using a Shimadzu GC-2014 gas chromatograph equipped with an FID, manufactured by Shimadzu Corporation, Kyoto-Japan. With a non-polar column of 30 m with a diameter of 250 μm and a film thickness of 0.25 μm. The conversion was calculated as the sum of the mass yields of dry gas (C_1_ - C_2_), liquefied petroleum gas (LPG, C_3_ - _4_), gasoline (C_5_ -216 °C), and coke fractions:

X = Y_drygas_ + Y_LPG_ + Y_gasoline_ + Y_coke_ (1)

where the yields of each lump (Y_i_) were defined as the total produced of this specific lump (m_i_) divided by the weight of the feedstock on a dry basis (m − m_water_):

Y_i_ = m_i_(m − m_water_)^−1^ × 100 (2)

The research octane numbers (RONs) of the gasoline cuts were calculated following the method reported by Anderson [54]. Mass balances (recoveries) in all the experiments of catalytic cracking closed to more than 95%. 

### 3.4. Coke Characterization

The coked catalysts were analyzed using a set of techniques already used in previous studies to discern the composition and location of the coke derived from the cracking reactions. Temperature-programmed oxidation (TPO) experiments were conducted to study the nature and quantify the deposited coke. The carbonaceous deposits were burned in a stream of oxygen diluted in nitrogen (1% *v*/*v*). Carbon monoxide (CO) and carbon dioxide (CO_2_) were produced as combustion products, which were subsequently converted into methane using a nickel catalyst in the presence of hydrogen. Mass balances were greater than 95 wt% in all cases. 

The coked catalysts were subjected to hydrofluoric acid (HF, 48 wt% in H_2_O, ≥99.99%) to obtain a soluble (S) coke fraction in dichloromethane (CH_2_Cl_2_, ACS reagent, ≥99.5%) and an insoluble (I) coke fraction. The soluble structures were the lightest fractions of coke and contained compounds that were not sterically blocked in the catalyst structure. The method involved grinding 200–300 mg of the coked catalyst, digesting it in HF, and extracting S coke using CH_2_Cl_2_. S coke was analyzed using a gas chromatograph (Shimadzu GC-2014) and the I coke fraction was analyzed by elemental analysis (Leco CHN628, LECO Corporation, St. Joseph, MI, USA). 

## 4. Conclusions

Utilizing a MAT-type laboratory reactor, this study meticulously examined the integration of residual cuts into the catalytic cracking process and its subsequent impact on catalyst activity. The co-processing of atmospheric residue (ATR) with vacuum gas oil (VGO) caused a synergistic effect that improved conversion, gasoline production, and gasoline quality as fuel. Notably, the CatY.0.20 catalyst, characterized by its superior mesoporosity and high concentration of strong Brønsted acid sites, demonstrated the highest conversion yields and exceptional efficiency in gasoline production, attaining yields exceeding 40 wt%.

The introduction of atmospheric residue significantly influenced the BET surface area and mesopore volume of the catalysts, leading to the accumulation of substantial concentrations of metals, nitrogen, and coke. Metals such as nickel, vanadium, and iron were found to deposit on the active sites of the catalyst, resulting in poisoning and pore blockage. Vanadium, with the highest concentration (4.22 ppm), was particularly detrimental, causing a pronounced loss of surface area, pore volume, and Brønsted acid sites. The ATR led to a reduction of more than 60% in the BET area of CatY.0.00 and up to 55% in CatY.0.20. Acidic properties were also adversely affected, with reductions of up to 70% in Brønsted acid sites and up to 50% in Lewis sites.

TPO profiles revealed that the coke produced from the residue was more challenging to oxidize, with combustion temperatures exceeding 580 °C, indicative of a more condensed and basic coke. This effect was more pronounced in the CatY.0.00 catalyst, which exhibited a maximum combustion peak at 650 °C. Co-processing VGO with ATR mitigated coke formation and reduced combustion temperatures, thereby facilitating the oxidation of coke trapped within the catalyst pores. FTIR analysis identified characteristic bands of aromatic and olefinic compounds in the coke, with ATR leading to higher intensities of aromatic coke, correlating with its high aromaticity and condensation. The VGO and VGO:ATR mixtures favored the formation of less condensed coke, with H/C ratios between 0.9 and 1.4, corresponding to aromatic species with one to four rings.

Despite coke being an undesirable byproduct, it is essential for maintaining the energy balance of the catalytic cracking process, providing the necessary fuel to achieve regeneration temperatures. Controlling coke formation is crucial to prevent premature catalyst deactivation and sustain the thermal efficiency of the process. This research presents a promising alternative for commercial applications in refining processes, offering significant innovations in catalytic cracking and waste management.

## Figures and Tables

**Table 1:** Feedstock properties.

Feedstock	VGO	ATR
°API	19.7	12.5
Aniline point (°C)	78.5	52.1
CCR (wt%)	0.43	5.9
Refractive index	1.4910	1.5523
Distillation curve (°C)		
Initial	272	294
10 vol.%	387	411
30 vol.%	420	473
50 vol.%	450	490
70 vol.%	487	518
95 vol.%	534	-
Final	582	-
SARA fractions (wt%)		
Saturated	47.4	9.80
Aromatic	50.0	35.1
Resin	2.10	26.8
Asphaltene	0.50	28.3
Nickel (ppm)	0.48	2.71
Vanadium (ppm)	0.97	4.22
Sodium (ppm)	0.83	3.41
Iron (ppm)	0.24	1.90
Sulfur (wt%)	1.12	4.63
Nitrogen (wt%)	0.28	1.40

**Table 2:** Textural, crystalline, and acidic properties of catalysts prepared from hierarchical zeolites.

	CatY.0.00	CatY.0.20
BET specific surface area, *S_BET_* (m^2^/g)	362	355
Mesopore-specific surface area, *S_meso_* (m^2^/g)	311	332
Total pore volume, *V_TP_* (cm^3^/g)	0.602	0.699
Micropore volume, *V_micro_* (cm^3^/g)	0.051	0.003
Mesopore volume, *V_meso_* (cm^3^/g)	0.551	0.669
$\mathrm{Average}\;\mathrm{mesopore}\;\mathrm{diameter},\;{\bar{d}}_{p}$(Å)	66.7	99.7
Zeolite content (wt%)	34.2	34.8
Crystallinity (%)	13	5
Unit cell size (Å)	24.22	24.19
Brønsted acidity (μmol Py/g)		
Weak	9	10
Medium	4	5
Strong	13	20
Lewis acidity (μmol Py/g)		
Weak	11	14
Medium	5	6
Strong	15	23

**Table 3:** Relative intensities of the FTIR bands corresponding to aromatic and olefinic coke deposited on both catalysts.

Signal	CatY.0.00			CatY.0.20		
	VGO	VGO:ATR	ATR	VGO	VGO:ATR	ATR
Aromatic band (1580 cm^−1^)	1.9	3.4	4.8	3.4	5.0	6.6
Olefinic band (1610 cm^−1^)	3.3	2.1	1.6	3.3	2.9	2.2

**Table 4:** Atomic H/C ratio of different hydrocarbons. Base structure: One aromatic ring, black; two aromatic rings, blue; three or more aromatic rings, red.

Structures	Molecular Formula	H/C Ratio
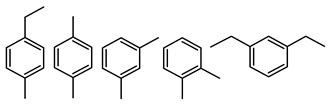	C_9_H_12_; C_8_H_10_; C_10_H_14_	1.20–1.40
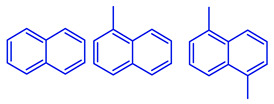	C_10_H_8_; C_11_H_10_; C_12_H_12_	0.80–1.00
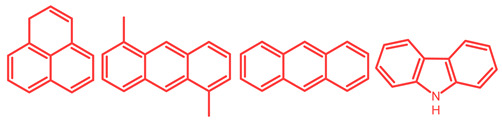	C_13_H_10_; C_16_H_14_; C_14_H_10_; C_12_H_9_N	0.80–0.90
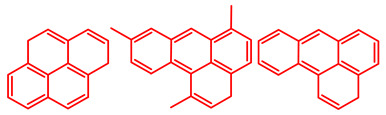	C_16_H_12_; C_20_H_18_; C_17_H_12_	0.70–0.90
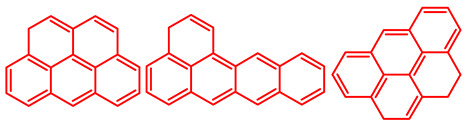	C_19_H_12_; C_21_H_14_; C_19_H_14_	0.60–0.70
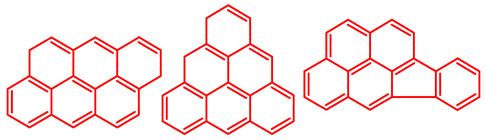	C_22_H_14_; C_22_H_12_	0.50–0.60
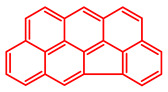	C_24_H_12_	0.50
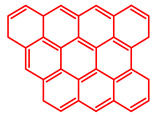	C_33_H_16_	0.48
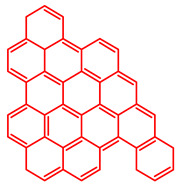	C_45_H_22_	0.49

**Table 5:** Elemental analysis of soluble and insoluble coke.

Insoluble Coke								
Feedstock	C (wt%)		H (wt%)		N (wt%)		H/C	
	CatY.0.00	CatY.0.20	CatY.0.00	CatY.0.20	CatY.0.00	CatY.0.20	CatY.0.00	CatY.0.20
VGO	12.1	12.3	0.58	0.61	0.61	0.90	0.57	0.59
VGO:ATR	12.3	11.8	0.60	0.52	0.82	1.10	0.58	0.53
ATR	13.4	12.9	0.41	0.48	2.50	2.85	0.36	0.44

**Table 6:** Properties of the fresh and coked catalysts used in the cracking of the feedstocks.

	CatY.0.00			CatY.0.20		
	Fresh	VGO	ATR	Fresh	VGO	ATR
BET specific surface area, *S_BET_* (m^2^/g)	362	215	129	355	269	202
$\mathrm{Average}\;\mathrm{mesopore}\;\mathrm{diameter},\;{\bar{d}}_{p}$(Å)	66.7	48	33	99.7	60	47
Brønsted acidity (μmol Py/g)						
Weak	9	2	1	10	5	4
Medium	4	2	3	5	3	4
Strong	13	8	2	20	10	6
Lewis acidity (μmol Py/g)						
Weak	11	8	6	14	10	7
Medium	5	4	3	6	4	3
Strong	15	14	12	23	18	14
Metals (wt%)						
Nickel	-	0.16	0.30	-	0.14	0.28
Vanadium	-	0.22	0.38	-	0.20	0.39
Sodium	-	0.18	0.34	-	0.19	0.36
Iron	-	0.12	0.19	-	0.11	0.16

## Data Availability

The data presented in this study are available in this article.
